# Short path molecular distillation of the essential oil from *Pinus roxburghii* oleoresin affords volatile fractions with powerful antioxidant and antimicrobial activities comparable with common synthetic agents and antimicrobials

**DOI:** 10.1016/j.heliyon.2025.e42282

**Published:** 2025-01-25

**Authors:** Muhammad Adnan Ayub, Hawraz Ibrahim M. Amin, Rameen Waseem, Kamaran Younis M. Amin, Muhammad Asif Hanif, Amjad Hussain, Kovan Dilawar Issa, Jorge Ramírez, Chabaco Armijos, Muhammad Zubair, Giovanni Vidari

**Affiliations:** a Department of Chemistry, University of Sahiwal, 57000, Sahiwal, Pakistan; b Department of Chemistry, College of Science, Salahaddin University-Erbil, Erbil, 44001, Iraq; c Department of Medical Biochemical Analysis, Cihan University-Erbil, Erbil, 44001, Iraq; d Department of Chemistry, College of Education, Salahaddin University-Erbil, Erbil, 44001, Iraq; e Department of Chemistry, University of Agriculture Faisalabad, Faisalabad, 38000, Pakistan; f Institute of Chemistry, University of Okara, Okara, 56300, Punjab, Pakistan; g Department of Medical Analysis, Faculty of Applied Science, Tishk International University, Erbil, 44001, Iraq; h Departamento de Química, Universidad Técnica Particular de Loja, Loja, 110107, Ecuador; i Department of Chemistry, Faculty of Science, University of Gujrat, Gujrat, 50700, Pakistan

**Keywords:** Chir pine oleoresin, Essential oil, Oil fractionation by molecular distillation, GC-MS analysis, Antioxidant and antimicrobial activities

## Abstract

The primary purposes of this work were to study the influence of the distillation temperature on the yield of the turpentine essential oil hydrodistilled from the *Pinus roxburghii* oleoresin and to obtain products with powerful bioactivities by short path molecular distillation of the oil. Our findings showed that the hydrodistillation temperature affected the yield as well as the antioxidant and antimicrobial properties of the essential oil: the highest yield (20.27 %) and bioactivities were obtained for the oil distilled at 180 °C. The oil was subsequently separated by short path molecular distillation into fractions and sub-fractions whose antioxidant activity was determined by the DPPH scavenging assay, the inhibition of linoleic acid peroxidation test, the FRAP assay, and the H_2_O_2_ scavenging assays. In addition, the antimicrobial activity of the fractions and sub-fractions against the bacteria *Pasteurella multocida, Staphylococcus aureus*, *Escherichia coli*, and *Bacillus subtilis*, and the fungal strains *Fusarium solani, Alternaria alternata, Aspergillus flavus*, and *Aspergillus niger*, was measured by the agar well diffusion method, the microdilution broth assay and the resazurin microtiter plate test. The antioxidant and antimicrobial activities varied between fractions and subfractions; however, their overall potency makes the *P. roxburghii* oleoresin a potential source of valuable natural antimicrobial products employable against various foodborne microbes and molds, as well as a source of preservative agents against food oxidation. Longifolene, 3-carene, α-pinene and β-pinene were identified by GC-MS analysis as the main constituents of the *P. roxburghii* turpentine essential oil and the most active fractions and sub-fractions. These terpenes were likely responsible for the bioactivity of the volatile mixtures.

## 1 Introduction

*Pinus roxburghii* Sargent, commonly known as “chir pine”, is a member of the Pinaceae family in the Gymnosperms. It is an evergreen cone-bearing tree with a height in the range from 30 to 55 m and a width in the range of 2.5–3.5 m [1]. Around 110–120 *Pinus* species exist, which are found in temperate areas of the northern hemisphere [2]. The Himalayan region, stretching from Afghanistan in the West to India, Pakistan, Bhutan and Nepal in the East, is home to the “chir pine”. The plant is typically found at altitudes from 450 to 2300 m [3]. Five species of Pinaceae, including *P. roxburghii*, grow in Pakistan, mainly in the rangelands of Baluchistan, North West Frontier and Punjab provinces, over an area of 1928 thousand acres [4]. The abundant presence of flavonoids, terpenoids, tannins, and xanthones in *P. roxburghii* makes the plant a well-known source of these specialized metabolites [5].

*P. roxburghii* is considered a herbicide, and an antiseptic, diaphoretic, diuretic, tonic, rubefacient, and vermifuge remedy [6]. Moreover, the use of the plant in Ayurvedic and Unani systems of medicine has a long history as an antilipidemic, intestinal antiseptic, antioxidant and spasmolytic remedy [7]. The tribal communities of North-West Frontier Province of Pakistan use *P. roxburghii* to treat bronchitis, ulcers, diaphoresis, itching, and ailments of the ears, eyes, throat, skin and blood [8]. In addition to possessing medicinal properties, the plant has many practical uses, providing charcoal, resin, pigment, and wood. The pine oleoresin, which is an abundant source of useful terpenes, consists of two major fractions, the volatile turpentine and the solid rosin. The turpentine essential oil isolated from *P*. *roxburghii* is commonly used as a paint thinner and as a solvent; moreover, it is appreciated medicinally, as a carminative, expectorant, analgesic and anthelmintic remedy. This volatile mixture contains 3-carene, longifolene and α-pinene as the main constituents, in addition to β-pinene, limonene, camphene, isopimaric and abietic acid [9]. Previous studies have determined the chemical composition and biological potential of the oleoresin essential oil tapped from *P. roxburghii* by chemical stimulants [10] or extracted by different distillation methods [11]. However, information is missing on the effect of distillation temperature on the oil yield and biological properties of fractions and sub-fractions obtained by further distillation of the essential oil.

The primary purpose of this work was to study the influence of the temperature on the hydrodistilled oil yield. Subsequently, the essential oil with the highest yield, EO_4_, was fractioned by molecular distillation. The antioxidant and antimicrobial properties of EO_4_ and the derived fractions and sub-fractions were determined by different *in vitro* assays, while their chemical composition was established by GC-MS analysis. Our interest in the antimicrobial and antioxidant properties of the essential oil (EO) and the fractions from the *P*. *roxburghii* oleoresin was due to the fact that EOs are considered among the most interesting nontoxic natural antioxidant and antimicrobial agents. They have demonstrated potential applications in the food industry which faces challenges from foodborne illnesses, due to microbial contamination, and food degradation caused by lipid oxidation. Natural remedies are considered to potentially reduce or replace antibiotics and synthetic additives, whose indiscriminate use has caused bacterial resistance and adverse effects on human health [12,13]. In fact, EOs prolong the food shelf-life, providing protection against oxidation and inhibiting the growth of pathogens.

Moreover, EOs and derived products have pleasant aromatic properties, which are highly valued to produce perfumes, cosmetics, food flavors and fragrances.

The various methods by which volatile fractions can be isolated from different plant parts strongly influence the product quality, the results’ accuracy, and the total analysis time and costs [14]. Hydrodistillation (HD) and steam distillation (SD) are the most common procedures for the extraction of essential oils [11]. Both processes are simple to perform and are cost-effective; moreover, they require the use of economic equipment and exclude organic solvents. They have, however, significant drawbacks, such as low extraction efficiency, loss of heat-sensitive volatile compounds due to long heating time, and substantial energy use. Modified and higher yield hydrodistillation processes, such as ultrasound- (or sono-) and microwave-assisted HD, are often used on a laboratory scale to isolate EOs [15]; however, they are generally not suitable on a large or industrial scale due to highly expensive operations and low product capacity [16].

Prevention of chemical oxidation and component deterioration of thermolabile compounds are beneficial characteristics of the ‘Short Path Molecular Vacuum Distillation (SPMVD)'or 'Molecular Distillation (MD)' method [17], that we used in the present work. The distillation apparatus is characterized by a distance of 10–50 mm between the evaporator and the condenser, which is equal to or smaller than the average free path of vaporized compounds, ensuring an extremely short residence time (1–10 s) of molecules in the evaporator, compared to hours on other conventional separation methods, and a collision-free mass transfer in the distillation region [[14], [15], [16]]. A short path also ensures that little compound is lost on the sides of the apparatus, improving the distillation yield. Moreover, due to the high vacuum (0.001–1 mbar) created in the system, SPMVD requires lower heating temperatures than traditional distillation techniques operating at atmospheric pressure. Thus, the reduced pressure and the low distillation temperatures help preserving the quality of thermally sensitive products, for example, several pharmaceutical and nutritional substances [17]. The SPMVD is also the preferred method for the separation of EOs [[18], [19], [20], [21], [22]] into different fractions based on the boiling points.

## 2 Experimental

### 2.1 Materials and methods

All the chemicals (highest grade) and biochemicals were purchased from Merck/Sigma-Aldrich (Milan, Italy).

#### 2.1.1 Collection of plant materials

Without using chemical stimulants, oleoresin was extracted by tapping the bark of *P. roxburghii* fully grown trees, collected in the Mansehra district of Khyber Pakhtunkhwa. Dr. Fahim Arshad, Associate Professor, Department of Botany, University of Okara, Pakistan, identified and verified the plant material. The oleoresin was air-dried in the shade, grinded manually, placed in polythene bags and stored at 4 °C until processing.

#### 2.1.2 Hydrodistillation

Fine-grained (80 mesh) oleoresin (300 g each batch) was hydrodistilled at 120 °C, 140 °C, 160 °C and 180 °C for 3 h. The resulting essential oils (EOs), labeled EO_1_, EO_2_, EO_3_, and EO_4_, respectively, were then dried with anhydrous Na_2_SO_4_, filtered, and stored at 4 °C for further analysis.

Each EO yield (EOY, w/w) was calculated by the following formula:

EOY (%) = [EO weight (g) / oleoresin weight (g)] × 100

The procedure was repeated three times and the average yield ± standard deviation (SD) was calculated.

#### 2.1.3 Fractionation of EO_4_

**Fig. 1:**
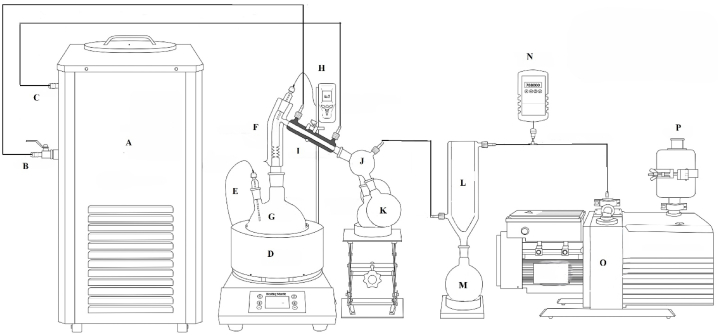
Schematic diagram of the used Short Path Molecular Distillation apparatus. A: cold liquid circulator; B: outlet; C: inlet; D: heating mantle; E: temperature probe; F: distillation head; G: sample flask; H: temperature probe; I: condenser; J: triple cow; K: fraction receiving flasks; L: cold trap; M: receiving flask; N: vacuum gauge; O: vacuum pump; P: air filter.

The short path molecular vacuum distillation (SPMVD) method was used as described previously [23], with minor changes, to separate EO_4_ into several fractions and sub-fractions. A schematic diagram of the equipment used by us is depicted in Fig. 1.

**Fig. 2:**
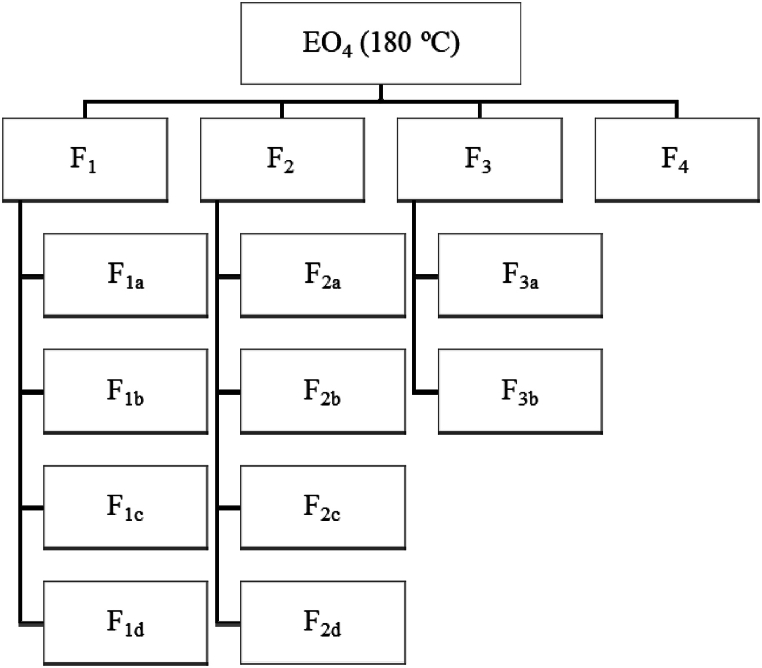
Flow chart of the short path molecular distillation process. I numeri non vanno messi al pedice (F1, non F_1_; F1 a, non F_1a_, F2 a, non F_2a_, etc.).

A glass column (2 m height × 3 cm internal diameter; 38 theoretical plates) was utilized for the batch vacuum distillation. Maintaining a constant flow rate and a constant pressure (76 mm Hg), four main fractions (F1-F4) were collected in the receiving flask by gradually increasing the pot temperature from 20 to 100 °C. Subsequently, the most abundant fractions F1-F3 were separated into the sub-fractions F1a-F1d, F2a-F2d, and F3a-F3b, respectively (Fig. 2), according to the boiling points (Table 1).

**Table 1:** Boiling point intervals and yields (mL) of the fractions obtained by molecular distillation of EO_4._

Fraction/Sub-fraction	Boiling point interval (°C)	Yield (mL)^#^
F1	62.5–68.6	25.50 ± 0.59^a^
F2	68.6–74.3	26.00 ± 0.51^a^
F3	74.3–76.5	13.00 ± 0.31^b^
F4	>76.5	4.50 ± 0.06^g^
F1 a	63–64.5	8.00 ± 0.12^d^
F1 b	64.5–66	11.00 ± 0.22^c^
F1 c	66–67.2	3.00 ± 0.05^i^
F1 d	>67.2	3.00 ± 0.03^i^
F2 a	69–70.6	6.25 ± 0.14^e^
F2 b	70.6–72	10.00 ± 0.20^c^
F2 c	72–73.5	6.00 ± 0.10^f^
F2 d	>73.5	3.50 ± 0.03^j^
F3 a	74,5-75	3.00 ± 0.01^h^
F3 b	>75	9.00 ± 0.11^d^

^a-j^Significant differences of p ≤ 0.05 between EOs hydrodistilled at different temperatures are indicated by different superscript letters.

^#^Mean value ± SD of 3 different experiments.

The volumes of EO_4_ and the separated fractions and sub-fractions were measured after drying with Na_2_SO_4_. Subsequently, each sample was analyzed by GC-MS and subjected to antioxidant and antimicrobial assays.

#### 2.1.4 Determination of the free radical scavenging/antioxidant activity

##### 2.1.4.1 DPPH assay

The 2,2-diphenyl-1-picrylhydrazyl (DPPH) radical-scavenging assay was performed on EO_4_ and the separated volatile fractions (paragraph 2.1.3.) following the procedure described in the literature [24] with minor adjustments. Each sample (100 mg in 1 mL of EtOH) was mixed with DPPH in 95 % MeOH (0.09 mM, 1 mL), followed by dilution with 95 % MeOH to a final volume of 4 mL. A solution of each sample (100 mg in 1 mL of EtOH), diluted with 95 % MeOH to a final volume of 4.0 mL, was used as the blank. The blank and the sample solutions were kept in complete darkness for 1 h at room temperature; subsequently, the absorbance of each sample at 515 nm was measured against the blank using a spectrophotometer (UV-2600i, Shimadzu, Kyoto, Japan). Butylated hydroxytoluene (BHT, 100 ppm) in 95 % MeOH was used as a reference standard. The lower was the absorbance of the sample (Abs_sample_), the higher was its free radical scavenging activity (FRSA).

The percent free radical scavenging activity (FRSA%) was calculated using the equation:

$$\text{FRSA}\%=\left({{\text{Abs}}_{\text{contro}}}_{\text{l}}-{\text{Abs}}_{\text{sample}}\;/\;{\text{Abs}}_{\text{control}}\right)\times 100$$

Where Abs_control_ was the absorbance of DPPH in 95 % MeOH (0.09 mM, 1.0 mL) added with 95 % MeOH (3.0 mL).

##### 2.1.4.2 FRAP assay

The total antioxidant content (TAC) of EO_4_, as well as the fractions and the sub-fractions obtained by fractional distillation (paragraph 2.1.3.), was evaluated using the slightly modified ferric ion reducing antioxidant power (FRAP) assay described in the literature [25]. Each sample (100 mg in 1 mL of EtOH), was separately mixed with a phosphate buffer (0.2 M, pH 6.7; 2.5 mL) and a potassium ferricyanide solution (1 % w/v; 2.5 mL). Test tubes were kept for 25 min at 50 °C in a water bath. Subsequently, a trichloroacetic acid solution (10 % w/v; 2.5 mL), followed by deionized water (2.5 mL), was added to each reaction mixture. Finally, a FeCl_3_ solution (0.1 % w/v; 500 μL) was added to each test tube. After an incubation of 30 min at room temperature, the absorbance of each reaction mixture was read at 700 nm with a UV/Vis spectrophotometer (UV-2600i, Shimadzu, Kyoto, Japan). The values of the total antioxidant content (TAC), expressed as mg/L of gallic acid equivalents (GAE), were calculated using a gallic acid calibration curve (0–100 mg/L) which showed linear regression (y = 0.021x - 0.0151, R^2^ = 0.99).

##### 2.1.4.3 Inhibition of linoleic acid peroxidation (LAPI) assay

The percent inhibition of linoleic acid peroxidation (% LAPI) was estimated according to a method in the literature [26]. EO_4_ and the separated fractions and sub-fractions (paragraph 2.1.3.) (50 μL each) were separately dissolved in 99.5 % EtOH (1 mL) and added with linoleic acid (2.5 %, v/v), 99.5 % EtOH (4 mL), and a sodium phosphate buffer (0.05 M, pH 7; 4 mL). The resulting mixtures were incubated at 40 °C for 175 h and then the daily extent of peroxidation was estimated by a colorimetric method. To each sample (0.2 mL), 75 % aqueous EtOH (10 mL), 30 % aqueous ammonium thiocyanate (0.2 mL), and FeCl_2_ (20 mM in 3.5 % HCl; 0.2 mL) were sequentially added. After stirring each mixture for 3 min, the absorbance was measured at 500 nm with a spectrophotometer (UV-2600i, Shimadzu, Kyoto, Japan). The lower was the absorbance of the test sample (Abs_sample_), the higher was the antioxidant capacity. The percent inhibition of linoleic acid peroxidation (% LPAI), was calculated with the following equation:

$$\%\;\text{LPAI}=\left[1-\left({\text{Abs}}_{\text{sample}}\text{ /}\;{\text{Abs}}_{\text{control}}\right)\right]\times 100$$

BHT (100 ppm) in 99.5 % EtOH was used as a reference standard.

##### 2.1.4.4 Hydrogen peroxide scavenging activity test

The hydrogen peroxide scavenging activities of EO_4_ and the separated fractions and sub-fractions (paragraph 2.1.3.) were determined spectrophotometrically using a slightly modified method described in the literature [27]. Each sample (600 μL) was separately added to a hydrogen peroxide solution (2 mM; 600 μL) in a phosphate buffer (pH 7.4). After incubation at 23 °C for 10 min, the absorbance of each mixture was measured at 230 nm. Ascorbic acid (100 mg/L) was used as a reference standard. The hydrogen peroxide scavenging activity (%) was calculated with the following equation:

% Hydrogen peroxide scavenging activity = [1− (A_1_/A_0_)] × 100

where A_1_ and A_0_ were the absorbances at 230 nm of the test sample and the control, respectively.

#### 2.1.5 Determination of the antimicrobial activity

##### 2.1.5.1 Microbial strains

The antimicrobial activities of EO_4_ and the fractions and sub-fractions derived by molecular distillation (paragraph 2.1.3.) were evaluated against the bacteria *Pasteurella multocida, Staphylococcus aureus,* *Escherichia* *coli,* and *Bacillus subtilis,* and the fungi *Fusarium solani, Alternaria alternata, Aspergillus flavus*, and *Aspergillus niger*. The strains were obtained from the University of Agriculture, Faisalabad, Pakistan.

##### 2.1.5.2 Agar well diffusion method

The antifungal and the antibacterial activities were measured using a slightly modified agar well diffusion method described in the literature [28]. Each strain culture, after growing overnight in the appropriate nutrient medium, was added into a flask containing nutrient agar (25 mL). Subsequently, the mixture was poured into moderate-sized Petri dishes and allowed to solidify at ambient temperature. Subsequently, each fungus and bacterium (10 and 20 μl each, respectively) was separately added to wells made in the solid mixtures with a sterilized borer. After an incubation period (24 h at 37 °C for the bacteria and 72 h at 28 °C for the fungi), the diameters (mm) of the zones of inhibition (ZOI) were measured. Ampicillin (1 mg/mL) and terbinafine (1 mg/mL) were used as the standard compounds in the antibacterial and antifungal assays, respectively.

##### 2.1.5.3 Resazurin micro-titer plate assay

The minimum inhibitory concentration (MIC) of EO_4_ and the separated fractions and sub-fractions (paragraph 2.1.3.) against each bacterial strain (paragraph 2.1.5.1.) was determined using a slightly modified 7-hydroxy-3H-phenoxazin-3-one 10-oxide (Resazurin) micro-titer plate assay described in the literature [29]. A stock solution of Resazurin dye was prepared by dissolving Resazurin sodium salt powder (0.50 g, Sigma-Alrich) into deionized H_2_O (100 mL). Subsequently, a working solution for Resazurin was prepared with 1:100 dilution of the stock solution with deionized H_2_O. Test sample (8.4 mg/mL) and the standard antibiotic amoxicillin (1 mg/mL) were separately dissolved in aqueous dimethyl sulfoxide (DMSO), and each resulting solution (10 % v/v, 10 mL) was carefully added to the first row of a 96-well plate. Nutrient Mueller Hinton (M − H) broth (50 μL) was added to the remaining wells. Using a multichannel pipette, two-fold dilutions were made so that 50 μL of each tested substance was present in each well at sequentially decreasing concentrations. Subsequently, to each well were added sequentially isosensitized nutrient broth of 3.3 × strength (30 μL), the Resazurin solution (10 μL), and a bacterial suspension (10 μL) to attain a nearly 5 × 10^5^ cfu/mL concentration. A cling film was wrapped loosely around each well to keep bacteria hydrated. Every plate contained a series of controls, including a positive control column, a column containing each solution except the test sample, a column containing each solution except the bacterium suspension (replaced by 10 μL of the nutrient broth), and a column containing the DMSO solution used as the negative control. The plates were prepared in triplicate and incubated at 37 °C for 24 h. Subsequently, the color change of Resazurin from purple to pink or colorless was evaluated visually. The lowest sample concentration causing the color change was considered the MIC of the test sample against the bacterial strain.

##### 2.1.5.4 Broth microdilution susceptibility test

The minimum inhibitory concentration (MIC) of EO_4_ and the separated fractions and sub-fractions (paragraph 2.1.3.) against each fungal strain (see paragraph 2.1.5.1.) was determined using the broth microdilution susceptibility assay described in the literature [30]. A 10 % (v/v) stock solution of each sample in DMSO was prepared, from which a series of dilutions in the range of 0.66–1351.08 μg/mL in a sterile culture medium was prepared in a 96 well micro-titer plate. The plate also contained a series of controls. Subsequently, each tested solution (20 μL) was added to each well, followed by Sabouraud Dextrose Broth (160 μL) and a standard fungus suspension (5 × 10^5^ cfu/mL, 20 μl). Terbinafine (1 mg/mL in 8 % DMSO) was used as a positive control. The plate was incubated at 30 °C for 48 h to grow the fungus. Broth clouding or separation of a cell layer at the bottom of a well signaled that the fungus had grown. The lowest concentration of the sample that completely inhibited the growth of the fungus was noted, and the average value ± SD of three assays was considered the MIC value.

#### 2.1.6 Gas chromatography-mass spectrometry (GC-MS) analysis

The GC–MS analyses of EO_4_ and the separated fractions and sub-fractions (paragraph 2.1.3.) were performed on a Shimadzu GC-2010 system [Shimadzu (Asia Pacific) Pte Ltd., Singapore] connected to a QP-2010 plus mass detector and equipped with a DB-5 capillary column (50 m × 0.25 mm, 0.25 μm of film thickness). The mass spectra were determined in the electron ionization mode (EI^+^) at 70 eV; filament emission current: 100 mA; ion source: 175 °C; transfer line: 240 °C; carrier gas: nitrogen at a flow rate of 1.5 mL/min; scan from 40 to 500 a.m.u. at a rate of 1 scan/sec. The mass spectra were acquired using the TurboMass software, version 5.4 (PerkinElmer Inc, Shelton, CT, USA). Each sample (1 μL of a 10 % solution (v/v) in *n*-hexane) was injected with a syringe into the gas chromatograph injection port. The column temperature was initially held at 60 °C for 3 min and then increased to 240 °C at a rate of 24 °C/min; the final temperature was held for 10 min. A commercial series of *n*-alkanes (C_7_-C_24_; Merck/Sigma-Aldrich, Milan, Italy) was injected after each sample under identical chromatographic conditions to determine the linear retention index (LRI_calcd_) of each sample components [31]. The components were identified (Table 2) by comparing the LRI_calcd_ values with those reported in the literature [[32], [33], [34]], and the mass spectra of the corresponding peaks in the gas chromatogram with the spectra contained in the Adams [32] and NIST [33] databases. Moreover, the identity of most oil components was confirmed by GC coelution with an authentic standard purchased from Merck/Sigma-Aldrich (Milan, Italy) or BenchChem (Pasadena, CA, USA). Each sample component was quantified following the methodology outlined in Ref. [35]. For this purpose, the response factor (RF_c_) of each component was calculated using the following equation:

$${\text{RF}}_{c}=A_{c}\times M_{\text{is}}\;/\;A_{\text{is}}\times M_{c}$$

**Table 2:** Chemical composition of EO_4_ and the most bioactive fractions and sub-fractions obtained by MD.

Components^**#,§,ǂ**;^	LRI_calcd_^**&**^	% abundance ±SD^†^								
		EO_4_	F1	F3	F4	F1 c	F2 b	F2 c	F3 a	F3 b
**Monoterpenoids**										
α-Thujene∗ 924		0.34± 0.00^a^	0.54 ± 0.02^a^	0.48 ± 0.01^b^	–	0.69 ± 0.03^a^	0.77 ± 0.01^a^	0.45 ± 0.00^b^	0.59 ± 0.03^c^	0.56 ± 0.02^a^
α-Pinene∗ (**1**) 933		26.89 ± 0.04^c^	53.1 ± 0.05^b^	31.99 ± 0.04^a^	5.19 ± 0.03^b^	69.12 ± 0.05^c^	66.48 ± 0.04^b^	53.49 ± 0.03^a^	58.01 ± 0.05^b^	46.93 ± 0.04^c^
Camphene∗ 949		0.4± 0.03^b^	0.68 ± 0.01^c^	0.6 ± 0.00^c^	0.16 ± 0.00^a^	0.75 ± 0.0^b^	0.89 ± 0.03^c^	0.66 ± 0.01^c^	0.78 ± 0.02^a^	0.76 ± 0.00^b^
Verbenene 971		0.61± 0.01^c^	1.06 ± 0.03^c^	0.81 ± 0.02^b^	0.62 ± 0.02^b^	0.64 ± 0.04^c^	0.8 ± 0.00^a^	0.81 ± 0.03^b^	0.85 ± 0.00^b^	0.84 ± 0.03^c^
β-Pinene∗ (**2**) 989		12.98± 0.02^b^	19.96 ± 0.04^b^	22.62 ± 0.03^a^	10.2 ± 0.01^c^	15.9 ± 0.00^a^	17.9 ± 0.02^b^	21.3 ± 0.02^a^	19.66 ± 0.01^c^	22.75 ± 0.05^a^
1,5,8-p-Menthatriene 1003		–	–	0.16 ± 0.01^a^	–	–	–	0.18 ± 0.02^c^	–	0.22 ± 0.02^b^
3- Carene∗ (**3**) 1010		43.89 ± 0.00^a^	15.96 ± 0.02^b^	38.97 ± 0.04^c^	38.2 ± 0.03^a^	10.01 ± 0.02^b^	11.96 ± 0.05^c^	17.54 ± 0.04^a^	15.86 ± 0.03^b^	25.85± 0.00^a^
*trans*-3-Caren-2-ol 1019		–	0.37 ± 0.00^b^	0.16 ± 0.04^a^	–	–	–	0.19 ± 0.01^a^	–	–
m-Cymene∗ 1024		0.47± 0.03^c^	0.35 ± 0.02^c^	0.53 ± 0.03^b^	0.85 ± 0.00^a^	–	–	0.29 ± 0.03^b^	0.38 ± 0.02^c^	0.38 ± 0.03^a^
p-Cymene∗ 1028		0.58± 0.04^b^	0.66 ± 0.01^a^	0.99 ± 0.00^b^	1.36 ± 0.02^c^	0.42 ± 0.00^a^	0.49 ± 0.04^b^	0.52 ± 0.00^b^	0.52 ± 0.04^a^	0.62 ± 0.01^c^
Limonene∗ 1030		0.38± 0.02^b^	–	0.57 ± 0.00^a^	0.72 ± 0.03^c^	–	–	–	–	0.29 ± 0.00^b^
Terpinolene 1096		1.00± 0.01^a^	–	0.76 ± 0.01^c^	1.98 ± 0.01^b^	–	–	–	–	–
Linalool∗ 1099		–	1.04 ± 0.01^b^	0.44 ± 0.01^a^	0.29 ± 0.00^c^	0.85 ± 0.03^a^	0.67 ± 0.01^c^	0.8 ± 0.00^a^	1.07 ± 0.02^c^	0.52 ± 0.03^b^
Thujol 1102		–	–	–	–	–	–	0.29 ± 0.01^c^	0.37 ± 0.03^b^	–
*cis*-Limonene oxide∗ 1138		0.2± 0.00^b^	0.96 ± 0.01^b^	0.34 ± 0.02^b^	0.37 ± 0.02^a^	0.5 ± 0.04^b^	–	0.74 ± 0.02^a^	0.65 ± 0.01^a^	0.26 ± 0.02^c^
*trans*-Limonene oxide∗ 1139		0.1± 0.04^b^	0.71 ± 0.00^c^	–	0.93 ± 0.0^a^	0.43 ± 0.02^c^	–	–	0.49 ± 0.00^a^	–
*trans*-Verbenol 1142		0.3± 0.03^c^	0.36 ± 0.03^c^	–	–	–	–	–	–	–
Pinocarvone 1161		–	0.86 ± 0.04^b^	0.17 ± 0.03^b^	–	0.36 ± 0.00^c^	–	0.51 ± 0.02^a^	0.37 ± 0.03^b^	–
Myrtenal∗ 1193		–	0.36 ± 0.02^c^	–	2.3 ± 0.03^b^	0.3 ± 0.01^a^	–	0.41 ± 0.03^b^	0.37 ± 0.01^c^	–
Verbenone∗ 1218		–	1.54 ± 0.01^b^	–	–	–	–	1.80 ± 0.01^a^	–	–
Bornyl acetate∗ 1284		0.1± 0.00^c^	–	–	0.98 ± 0.01^b^	–	–	–	–	–
**Sesquiterpene Hydrocarbons**										
α-Longipinene (**4**) 1350		0.75± 0.02^c^	1.25 ± 0.04^a^	–	3.4 ± 0.04^c^	–	–	–	–	–
α-Ylangene 1366		0.21± 0.01^a^	–	–	–	–	–	–	–	–
Longicyclene 1372		0.22± 0.03^a^	–	–	1.4 ± 0.03^a^	–	–	–	–	–
Sativene 1392		0.2± 0.01^b^	0.2 ± 0.01^c^	–	0.63 ± 0.01^b^	–	–	–	–	–
Longifolene∗ (**5**) 1407		8.88± 0.03^b^	–	0.38 ± 0.01^c^	27.5 ± 0.02^b^	–	–	–	–	–
*trans*-Caryophyllene∗ 1416		0.7± 0.04^c^	–	–	1.99 ± 0.03^a^	–	–	–	–	–
**Oxygenated Sesquiterpenoids**										
Caryophyllene oxide 1580		0.78± 0.00^a^	–	–	0.91 ± 0.01^c^	–	–	–	–	–
Total		99.98	99.96	99.97	99.98	99.97	99.96	99.98	99.97	99.98

^a-c^ Significant differences of p ≤ 0.05 between EOs extracted by HD at different temperatures are indicated by different superscript letters.

†% Abundances and standard deviations (SD) were calculated as the mean of three distinct experiments. The compounds were identified.

#by comparison of the LRI_calc_ with the literature [[32], [33], [34]].

§by comparing the mass spectrum of each peak with the spectra contained in reference databases [32,33].

∗by GC coelution with an authentic standard purchased from Merck/Sigma-Aldrich (Milan, Italy) or Bench2Chem (Pasadena, CA, USA).

ǂThe bold numbers in brackets refer to the structures depicted in Fig. 12.

&LRI_calc_ = calculated linear retention index on a HP-5MS capillary column relative to a homologous series of standard *n*-alkanes (Merck/Sigma-Aldrich, Milan, Italy, No. CE 203-777-6), from *n*-heptane (C_7_) to *n*-tetracosane (C_24_), which were injected after each sample under the same experimental conditions [31].

where A_c_ and A_is_ were the integrated peak areas of an authentic sample of the component and *n*-undecane, used as an internal standard, respectively, while M_c_ and M_is_ were the concentrations (molarity) of the corresponding solutions injected into the GC instrument. Subsequently, a solution of a sample was injected into the GC system, and the % of each component was calculated using the equations (a) and (b):

Corrected area of a component = integrated peak area/RF_c_ of the component (a)

Percentage (%) of a component = (corrected area of the component/total corrected area of all components) × 100 (b)

For a component with an unavailable standard, the RF_c_ was considered equal to 1.

#### 2.1.7 Statistical analysis

Each experiment was conducted in triplicate, and the mean ± standard deviation (SD) was calculated. Statistical analysis of the data was performed by Analysis of Variance (ANOVA) using STATISTICA 5.5 (Stat Soft Inc., Tulsa, OK, USA) software. Results were considered statistically significant at p ≤ 0.05.

## 3 Results and discussion

### 3.1 Essential oil yield (EOY)

**Fig. 3:**
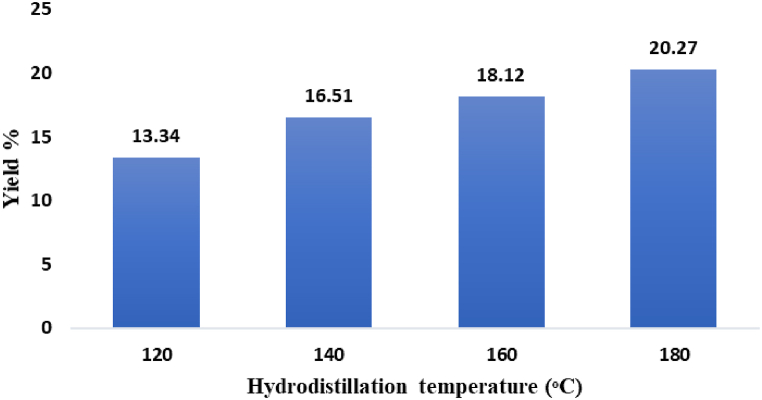
Yields of the EOs hydrodistilled from *P*. *roxburghii* oleoresin at different temperatures.

The % yields (w/w) of the EOs isolated from the *P. rosburghii* oleoresin by increasing the hydrodistillation temperature, are shown in Fig. 3. The lowest yield (13.34 ± 0.17 %) was observed for the oil (EO_1_) isolated at 120 °C, while the highest one (20.27 ± 0.30 %) was obtained for the oil (EO_4_) hydrodistilled at 180 °C. The last value is consistent with the yield of 26.7 % reported in the literature for an EO from a *P. rosburghii* oleoresin [9]. Instead, hydrodistillation of needles, bark, and stems of *P. roxburghii* afforded EOs in significantly lower yields, in the range of 0.11–1.83 % [2,36,37].

The dependence of the yield of the EOs on the distillation temperature may be due to an enhanced diffusion and an increased solubility of the oil in water at higher temperatures. Elevated temperature expedites the diffusion of the components of an EO in plant tissues, facilitating their transfer from the cells where they are synthesized or stored to the extraction medium. Furthermore, increased solubility can lead to increased EO extraction from the plant.

### 3.2 Fractionation of EO_4_

A batch (75 mL) of EO_4_ was separated by short path molecular vacuum distillation into four fractions (F1-F4), and the most abundant fractions F1-F3 were further separated into sub-fractions, F1 a-F1 d, F2 a-F2 d, and F3 a-F3 b (Fig. 2 and Table 1). The boiling point intervals and the yields (mL) of the different fractions and sub-fractions are reported in Table 1. The total recovery yield of F1-F4 was 92 % with respect to EO_4_, while the overall yield of the subsequent distillations was 97.3 %. Loss of products was due to the inevitable escape of volatile components during the distillations.

### 3.3 Antioxidant/antiradical properties of the volatile fractions isolated from the *Pinus roxburghii* oleoresin

#### 3.3.1 DPPH free radical scavenging activity

**Fig. 4:**
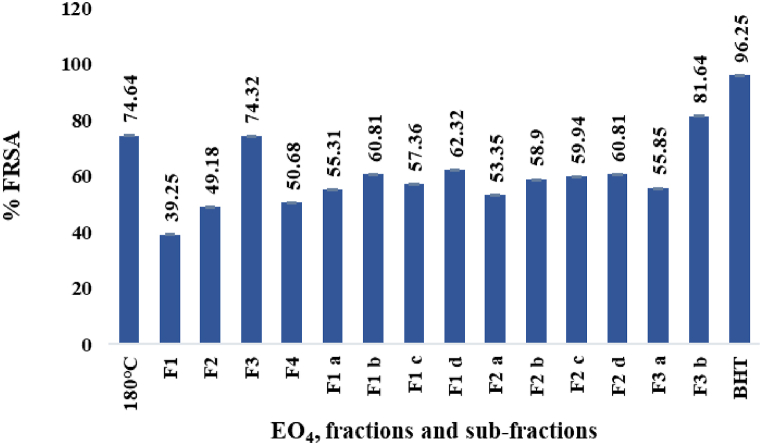
% FRSA in the DPPH test.

The percent free radical scavenging activity (% FRSA) in a DPPH test of EO_4_ (180 °C), the derived fractions and sub-fractions (paragraphs 2.1.3. and 3.2.) and the standard BHT, are shown in Fig. 4.

Among the EO_s_ hydrodistilled from *P. roxburghii* oleoresin at different temperatures (paragraph 2.1.2), EO_4_ displayed the highest free radical scavenging activity (74.64 %), that was almost maintained in the fraction F3 (74.32 %), while it significantly increased in the sub-fraction F3 b (81.64 %). These high values were consistent with the % FRSA of the EOs hydrodistilled from needles (50 %), bark (85 %) and wood (82 %) of *P*. *roxburghii* [38].

#### 3.3.2 FRAP assay/total antioxidant content

**Fig. 5:**
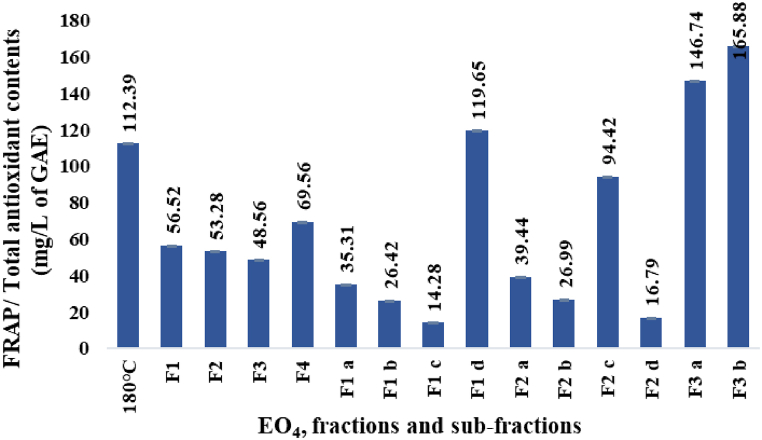
FRAP assay/total antioxidant content.

The total antioxidant content (TAC) of EO_4_ and the derived fractions and sub-fractions (paragraph 2.1.3.) are displayed in Fig. 5. The values are expressed as mg/L of gallic acid equivalents (GAE) and were calculated using a calibration curve showing linear regression (y = 0.021x - 0.0151, R^2^ = 0.99).

The overall antioxidant contents were in the range of 14.28–165.88 mg/L of GAE (Fig. 5). Among the four EOs hydrodistilled from the oleoresin (Fig. 3), EO_4_ displayed the highest antioxidant content (112.39 mg/L of GAE). The activity increased considerably in the sub-fractions F3 a (146.74 mg/L of GAE) and F3 b (165.88 mg/L of GAE), while decreased significantly in the sub-fractions F2 d (16.79 mg/L of GAE) and F1 c (14.28 mg/L of GAE).

#### 3.3.3 Per cent inhibition of linoleic acid peroxidation

**Fig. 6:**
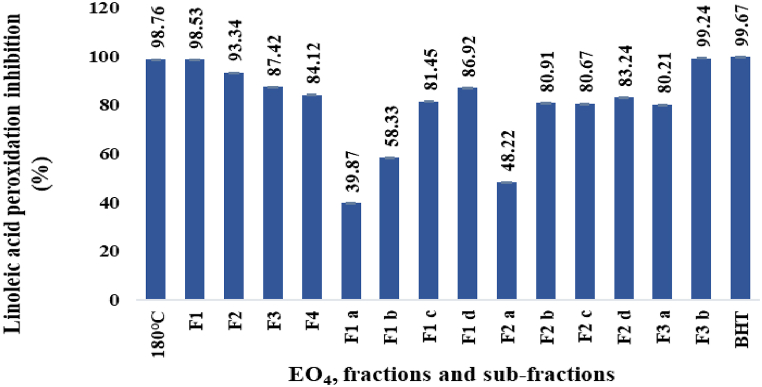
% Inhibition of linoleic acid peroxidation.

The % inhibition of linoleic acid peroxidation (% LAPI) by EO_4_, the derived fractions and sub-fractions (paragraph 2.1.3.) and the standard BHT (100 ppm), are shown in Fig. 6. In general, all samples inhibited the linoleic acid peroxidation (LAP) significantly.

Among the EOs distilled from the *P. roxburghii* oleoresin (Fig. 3), EO_4_ displayed the greatest capability to suppress the linoleic acid peroxidation (98.76 %). The activity was maintained in the fraction F1 (98.53 %) and increased in the sub-fraction F3 b (99.24 %), whose value is comparable with the reference standard BHT (99.67 %). Moreover, it is noteworthy that in each group of sub-fractions, the antioxidant activity increased with the boiling point.

#### *3.3.4* *H*_*2*_*O*_*2*_*scavenging activity*

**Fig. 7:**
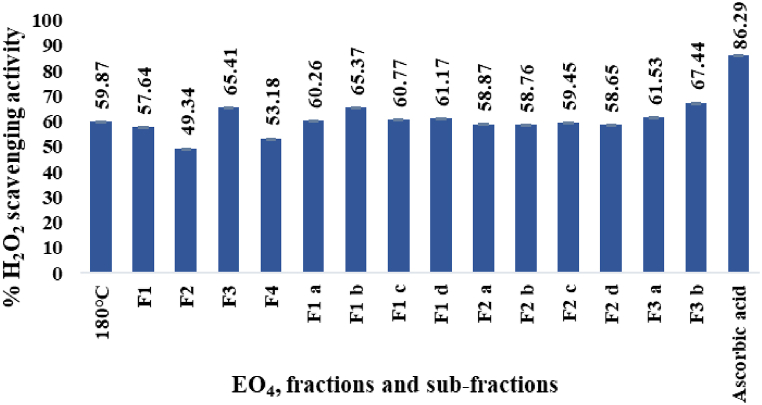
% H_2_O_2_ scavenging activity.

The percent hydrogen peroxide scavenging activities (HPSA) determined for EO_4_, the derived fractions and sub-fractions (paragraph 2.1.3.) and the standard ascorbic acid (100 mg/L), are displayed in Fig. 7. In general, the effects were only moderate, in the range of 49.34 to 67.44 %, and were lower than those shown by ascorbic acid.

Among the EOs hydrodistilled from the *P. roxburghii* oleoresin (Fig. 3), EO_4_ exhibited the highest H_2_O_2_ scavenging activity (59.87 %). On the other hand, F3 was the most potent (65.41 %) among the fractions derived from EO_4_ by molecular distillation, while F2 was the least active (49.34 %). The hydrogen peroxide scavenging activity increased in the sub-fraction F3 b (67.44 %), while it remained low in the sub-fractions F2 a-d (from 58.65 to 59.45 %).

Overall, the different chemical compositions of the EOs hydrodistilled from the *P. roxburghii* oleoresin, and the fractions and sub-fractions obtained by subsequent molecular distillation, might cause differences in the observed antioxidant properties. The highest activity shown by EO_4_ in all the four antioxidant assays was probably due to the high content of the monoterpene hydrocarbons α-pinene (26.89 %), β-pinene (12.98 %), and 3-carene (43.89 %), as well as some sesquiterpene hydrocarbons, especially longifolene (8.88 %) (see Table 2). This finding is in accordance with other studies of the essential oil of *P. roxburghii* [39]. Terpenoids were also responsible for the powerful antioxidant effects of the fractions F1, F3, F4 and the sub-fractions F3 a and F3 b, that were significantly stronger than the parent oil EO_4_.

### 3.4 Antimicrobial activity

The well diffusion, the broth microdilution MIC, and the colorimetric resazurin microtiter plate assays were used to measure, semi quantitatively, the *in vitro* activity against different microbial isolates of EO_4_ (180 °C) and the fractions and sub-fractions derived from EO_4_ by molecular distillation (paragraph 2.1.3.).

#### 3.4.1 Antibacterial activity

**Fig. 8:**
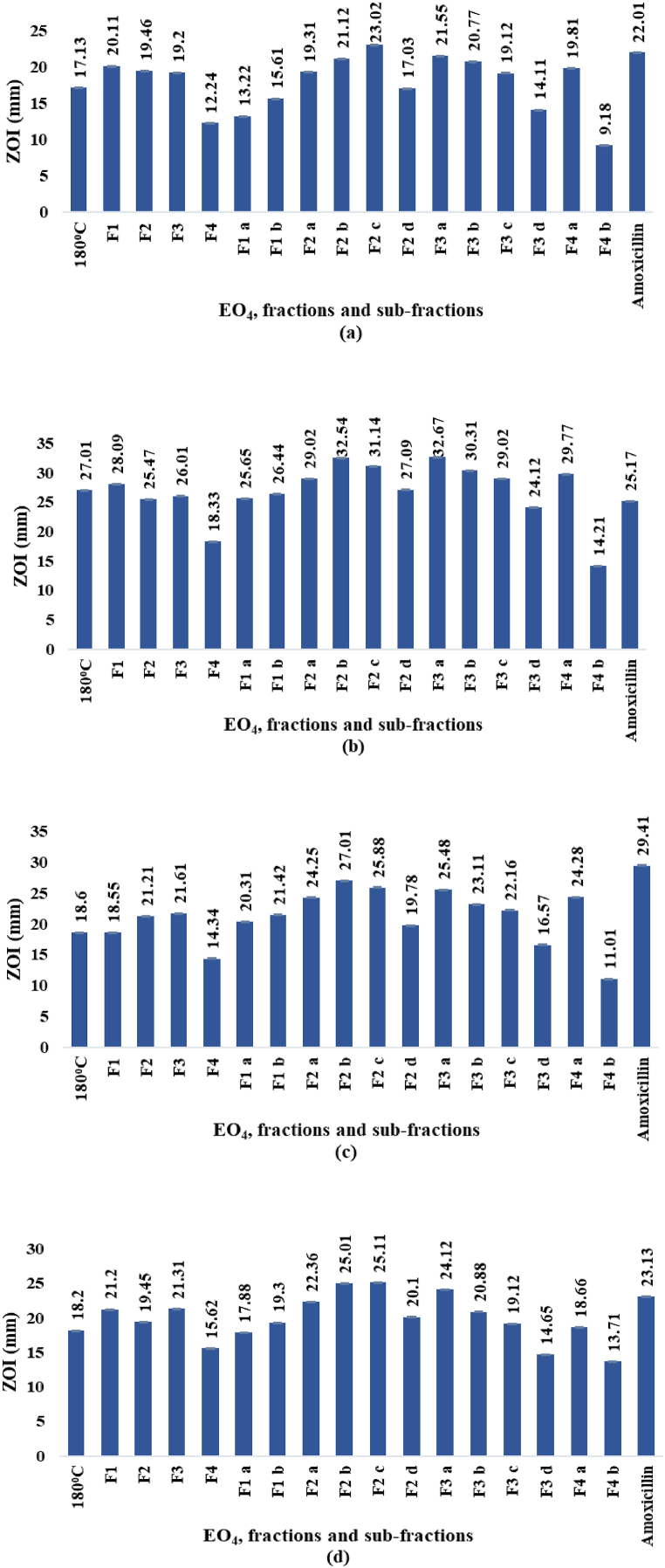
a–d. ZOI diameters (mm) against *E. coli* (a), *S. aureus* (b), *P. multocida* (c), and *B. subtilis* (d), respectively.

**Fig. 9:**
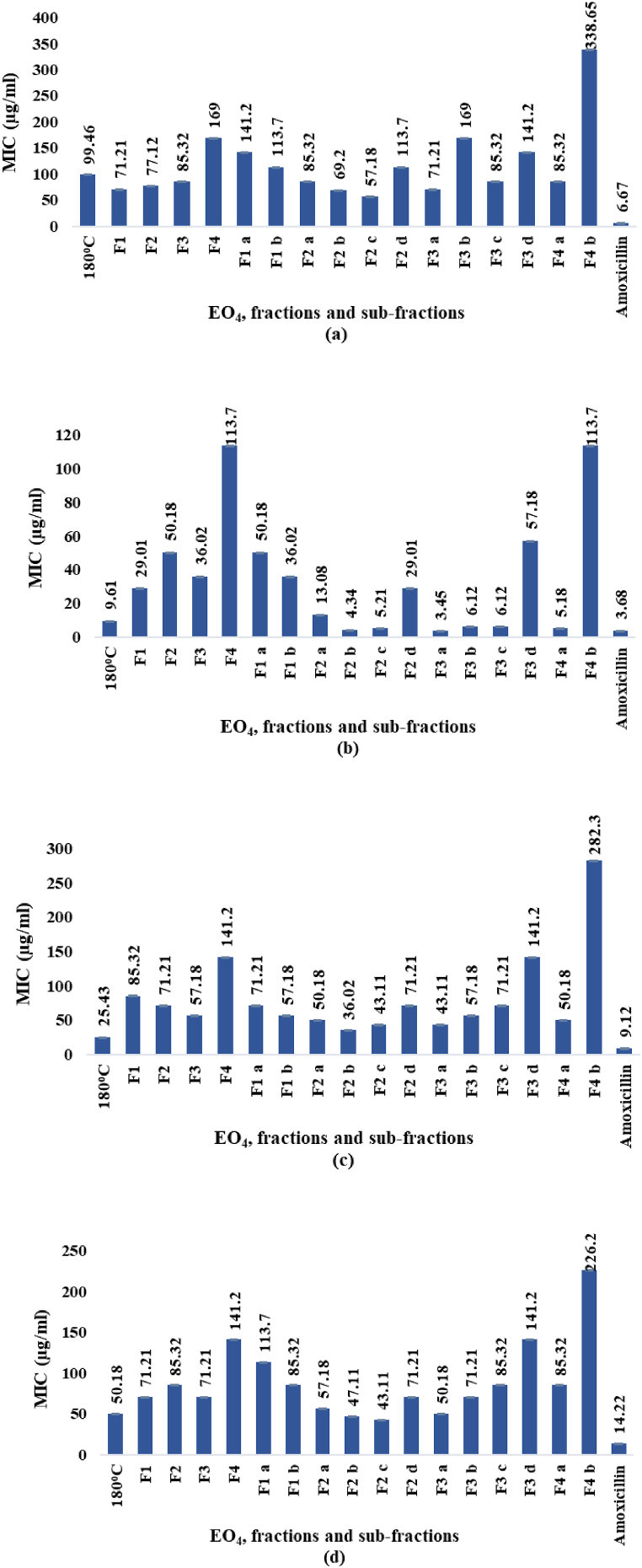
a–d. MICs (μg/mL) against *E. coli* (a), *S. aureus* (b), *P. multocida* (c), and *B. subtilis* (d), respectively.

The diameters of inhibition zones (ZOI) and the MICs determined by *in vitro* tests against *E. choli, S*. *aureus, P. multocida,* and *B*. *subtilis* for EO_4_, and the fractions and sub-fractions obtained by molecular distillation (paragraph 2.1.3.), are displayed in Fig. 8, Fig. 9d. The ZOI diameters and the MIC values ranged from 9.18 to 32.67 mm and 3.45–338.65 μg/mL, respectively. In comparison, the values observed for the standard antibiotic amoxicillin (1 mg/mL) ranged from 22.01 to 29.41 mm and 3.68–14.22 μg/mL, respectively.

Among the EOs (Fig. 3) hydrodistilled from the oleoresin, EO_4_ displayed the highest activity against the four bacterial strains, as indicated by the ZOI diameters (Fig. 8a–d), which ranged from 17.13 mm against *E. coli* to 27.01 mm against *S. aureus*, and the lowest MICs (Fig. 9a–d), which ranged from 9.61 μg/mL against *S. aureus* to 99.46 μg/mL against *E. coli*. Overall, the antibacterial effects of EO_4_, especially against *S. aureus*, were almost as powerful as the reference antibiotic amoxicillin, at the concentration used in the assay (1 mg/mL). Among the fractions, F1 exhibited the strongest effects against *E. coli* and *S. aureus*, while F3 was the most active against *B. subtilis* and *P. multocida*. The sub-fraction F2 c showed the highest antibacterial effects against *B. subtilis* and *E. coli*, while F3 a and F2 b exhibited the strongest antibacterial activity against *S. aureus* and *P. multocida*.

The greater activity of F3 a than amoxicillin against *S. aureus* is remarkable, since there is a continuous search for novel antibacterial products, especially from nature, to fight this pathogen with higher efficacy than the commonly used compounds or having a different mechanism of action. In fact, the gram-positive bacterium *S. aureus* is frequently found in the upper respiratory tract and on the skin. Although it usually acts as a commensal of the human microbiota, it can also become an opportunistic pathogen, being a common cause of skin infections including abscesses, respiratory infections such as sinusitis, and life-threatening illnesses such as pneumonia, meningitis, osteomyelitis, endocarditis, toxic shock syndrome, bacteremia, and sepsis. Moreover, *S. aureus* is still one of the five most common causes of hospital-acquired infections and is often the cause of wound infections following surgery. Up to 50,000 deaths each year in the U.S. is linked to staphylococcal infections. Indeed, *S. aureus* is a worldwide problem in clinical medicine, because it is one of the leading pathogens for deaths associated with antimicrobial resistance and the emergence of antibiotic-resistant strains, such as methicillin-resistant *S. aureus* (MRSA) (cited from Staphylococcus aureus - Wikipedia; accession date: 1 September 2024).

#### 3.4.2 Antifungal activity

**Fig. 10:**
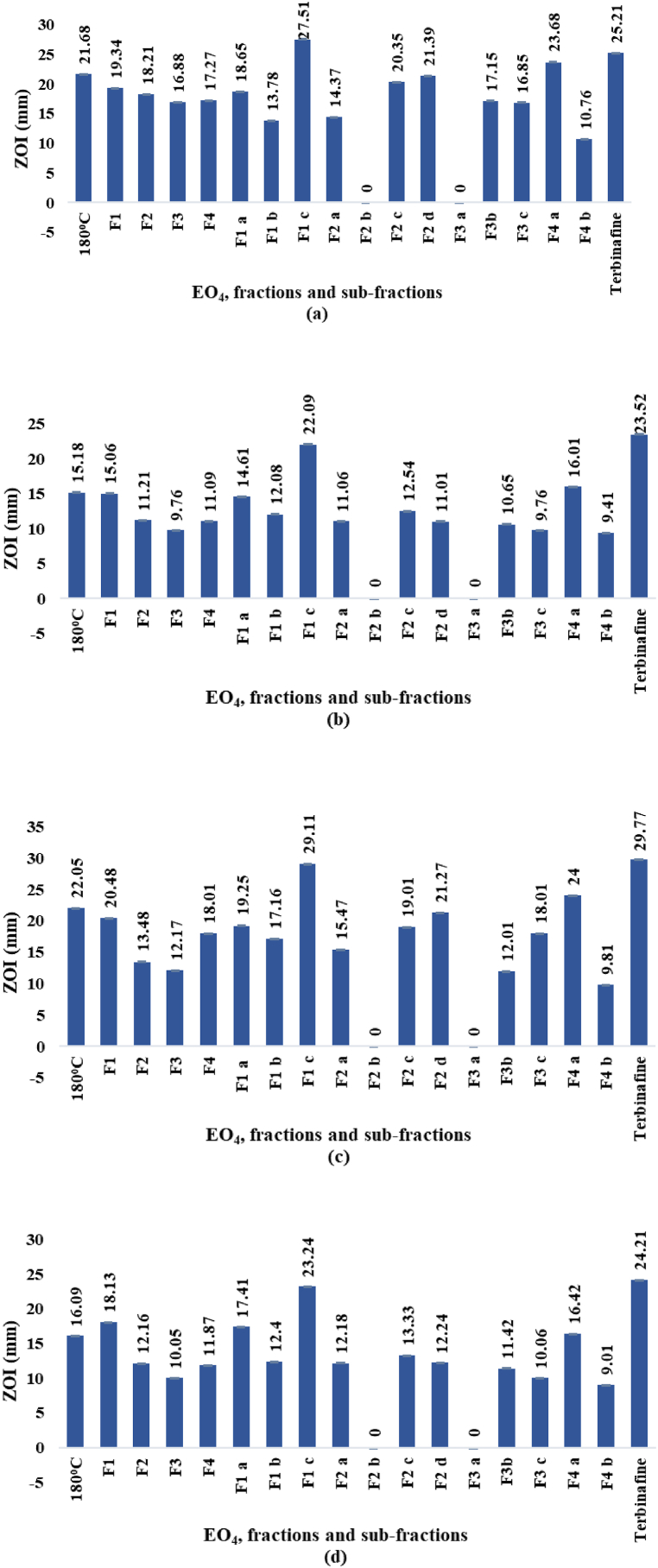
a–d. ZOI diameters (mm) against *F. solani* (a), *A. niger* (b), *A. alternata* (c), and *A. flavus* (d), respectively.

**Fig. 11:**
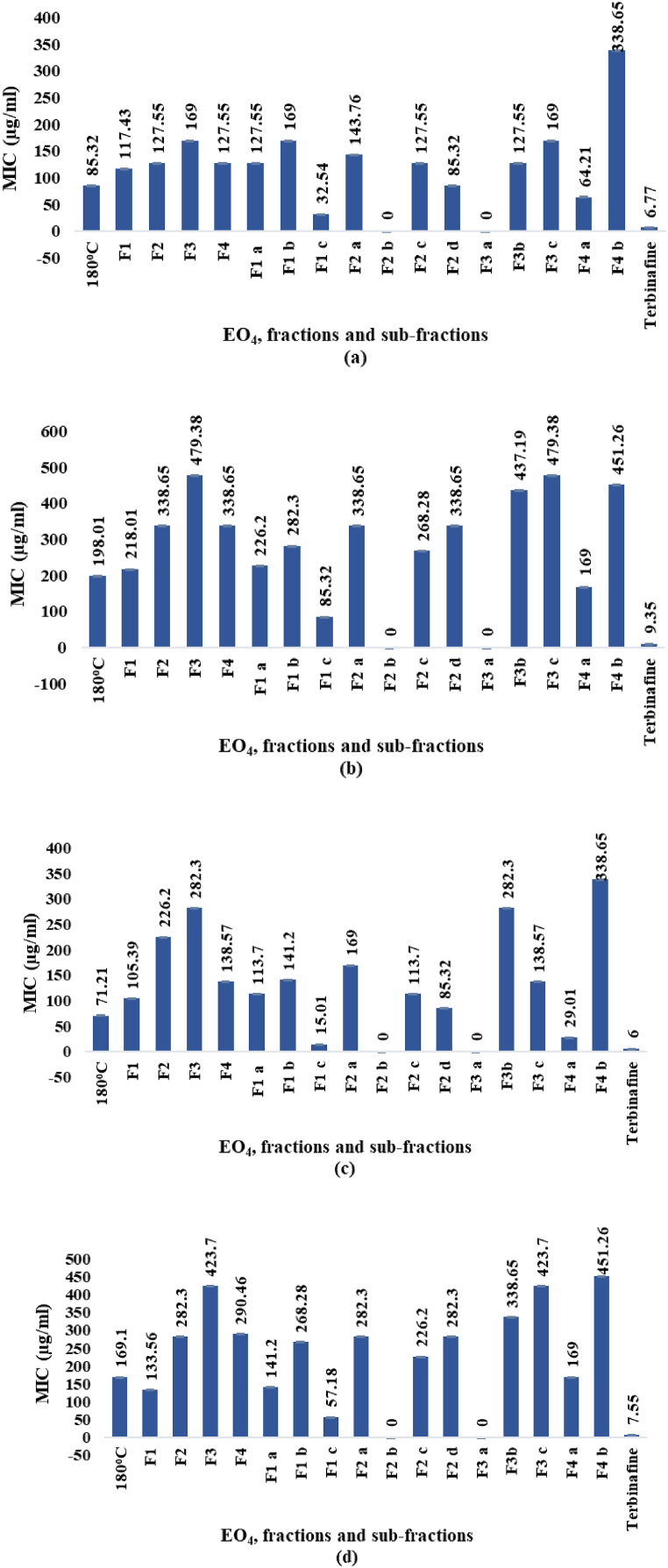
a–d. MICs (μg/mL) against *F. solani* (a), *A. niger* (b), *A. alternata* (c), and *A. flavus* (d), respectively.

The diameters of inhibition zones (ZOI) and the MICs determined *for* EO_4_ and the derived fractions and sub-fractions (paragraph 2.1.3.) by *in vitro* tests against *F*. *solani, A*. *niger, A*. *alternata,* and *A*. *flavus*, are shown in Fig. 10a–d and 11a-d, respectively. The ZOI diameters (mm) and the MICs (μg/mL) ranged from 9.01 to 29.11 mm and 15.01–479.38 μg/mL, respectively. In comparison, the values shown by the reference antifungal agent terbinafine (1 mg/mL) in 8 % DMSO ranged from 23.52 to 29.77 mm and 6.00–9.35 μg/mL, respectively.

Among the EOs (Fig. 3) hydrodistilled at different temperatures from the oleoresin of *P. roxburghii*, EO_4_ displayed the highest antifungal activity. *A. alternata* and *F. solani* were the most sensitive fungi to EO_4_. However, EO_4_ was less potent than the reference antifungal agent terbinafine at the concentration used in the text. Upon fractionation by MD, the antifungal activity was mainly exhibited by the fraction F1, while decreased considerably for the high boiling fraction F3. Upon molecular distillation of F1, the antifungal effects against the four strains increased significantly for the sub-fraction F1 c. In fact, the ZOI diameters as well as the MICs determined for F1 c were comparable with those of terbinafine. Especially sensitive to the antifungal effects of F1 c was *A. alternata,* which is the causative agent of leaf spots, rots, and blights on many plant parts, as well as upper respiratory tract infections and asthma in humans with compromised immunity.

In summary, EO_4_ and some fractions and sub-fractions obtained by molecular distillation of the oil, exhibited from moderate to excellent antifungal properties against the four tested fungal strains.

The zero values assigned to the activities of F2 b and F3 a in Fig. 10, Fig. 11 indicated that no significant value could be measured, and the two fractions were considered inactive.

Overall, the different antimicrobial activities of EO_4_ and the active fractions and sub-fractions obtained by MD might depend on the different chemical compositions (Table 2). Monoterpene and sesquiterpene hydrocarbons, especially α-pinene, β-pinene, 3-carene, and longifolene, whose relative abundance varied in the different volatile mixtures, were possibly the major contributors to the antimicrobial effects [[39], [40], [41], [42], [43]], which are not attributable to a single mechanism [44]. As regard the activity of terpenoid hydrocarbons, due to their hydrophobicity, they may penetrate the bacterial cell wall, eventually disrupting the lipid structure and causing protein dysfunction [45,46]. These effects ultimately result in microbes’ cell lysis, cytoplasmic leakage, and death [47,48].

### 3.5 GC-MS analysis

The chemical compositions of EO_4_ (Fig. 3), the fractions F1, F3, F4 and the sub-fractions F1 c, F2 b, F2 c, F3 a, and F3 b (Fig. 2), which showed the highest antioxidant (paragraph 3.3.) and antimicrobial (paragraph 3.4.) activities, were established by GC-MS analysis. The data are shown in Table 2. Twenty-one compounds with an abundance ≥0.1 % were identified in EO_4_, the majority of which were monoterpenes (87.54 %), followed by sesquiterpenes (10.96 %), while oxygenated sesquiterpenoids and monoterpenoids only accounted for 0.78 and 0.70 %, respectively, of the total composition of the oil. These findings were consistent with the literature, confirming the high abundance of monoterpenes in the EOs isolated from *P. roxburghii* [2], except for the oil extracted from the bark of the plant grown in Nepal, in which sesquiterpenes were predominant [49].

3-Carene (43.89 %), α-pinene (26.89 %), β-pinene (12.98 %), longifolene (8.88 %) and α-longipinene (0.75 %) were the most abundant mono- and sesquiterpenes in EO_4_. α-Thujene, terpinolene, linalool, cymene, and *trans*-caryophyllene were minor components of EO_4_, as well as fractions and subfractions.

Twenty, sixteen and twenty compounds were identified in the fractions F1, F3 and F4, respectively, while only 12, 8, 18, 14, and 12 compounds were identified in the sub-fractions F1 c, F2 b, F2 c, F3 a, and F3 b, respectively. Monoterpenes, mainly 3-carene, α-pinene, and β-pinene, were the predominant components of F1, F3 and F4; however, their amounts varied in the different fractions, due to the different volatility. Moreover, the percentages of sesquiterpene hydrocarbons (34.92 %) and oxygenated monoterpenes (4.87 %) increased significantly compared to monoterpenes (59.28 %) in the highest boiling fractions F4, in contrast with the relative abundances of the same terpenoid families in the parent oil EO_4_. This finding was due to the high boiling points of sesquiterpenes and oxygenated monoterpenoids, which required higher distillation temperatures than monoterpenes; furthermore, some terpenoid hydrocarbons may have been oxidized at high temperatures.

Similar considerations can be made regarding the composition of the sub-fractions F1 c, F2 b, F2 c, F3 a, and F3 b. In fact, monoterpene hydrocarbons, followed by oxygenated monoterpenoids, were the main components. 3-Carene, α-pinene, and β-pinene largely predominated among the monoterpenes, while limonene oxide and linalool were the main oxygenated monoterpenoids. Their distribution in the different sub-fractions can be attributed to the different volatility combined with the different temperatures used in the molecular distillations.

**Fig. 12:**
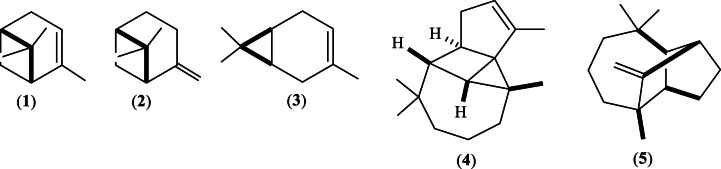
Structures of α-pinene (**1**), β-pinene (**2**), 3-carene (**3**), α-longipinene (**4**), and longifolene (**5**).

Some characteristic components of the volatile mixtures extracted from the oleoresin of *P. roxburghii* are depicted in Fig. 12. Only the relative stereochemistry is shown.

## 4 Conclusions

In summary, we have found that the yield of the essential oil extracted from the *P. roxburghii* oleoresin significantly improved by increasing the hydrodistillation temperature. The highest value was obtained for the oil (EO_4_) hydrodistilled at 180 °C. Separation of this oil by molecular distillation afforded a few volatile fractions and sub-fractions, rich in terpenoids, whose antiradical/antioxidant effects and antimicrobial activities increased compared to the parent oil. This finding clearly demonstrated the importance of fractionating an essential oil by molecular distillation to obtain products with more powerful antioxidant and antimicrobial properties. For example, the activity of the sub-fraction F3 against *S. aureus* was higher than the antibiotic amoxicillin at the concentration used in the assay, while the antifungal activity of F1 c and F4 a were comparable with those determined for the antifungal agent terbinafine.

In conclusion, the potent antimicrobial and antioxidant properties shown by EO_4_ and some of the fractions and sub-fractions obtained by molecular distillation, make the *P. roxburghii* oleoresin a potential source of natural antimicrobial products against various foodborne microbes and molds, as well as a source of effective preservative agents against food oxidation. These products may thus become an alternative to the commonly used synthetic preservatives that often cause allergic reactions and a number of health problems [50].

## CRediT authorship contribution statement

**Muhammad Adnan Ayub:** Writing – original draft, Data curation, Conceptualization. **Hawraz Ibrahim M. Amin:** Project administration, Investigation, Conceptualization. **Rameen Waseem:** Writing – review & editing, Writing – original draft. **Kamaran Younis M. Amin:** Validation, Project administration. **Muhammad Asif Hanif:** Project administration, Methodology. **Amjad Hussain:** Resources. **Kovan Dilawar Issa:** Funding acquisition. **Jorge Ramírez:** Funding acquisition, Formal analysis. **Chabaco Armijos:** Validation, Funding acquisition, Data curation. **Muhammad Zubair:** Writing – original draft. **Giovanni Vidari:** Writing – review & editing, Data curation, Conceptualization.

## Data availability statement

All data generated or analyzed during this study are available by request to the corresponding author M.A.A. (adnanayub@uosahiwal.edu.pk).

## Ethical approval

Not required.

## Ethics statement

Not applicable.

## Funding

This research is part of a research project funded by the 10.13039/501100004681Higher Education Commission (HEC), Islamabad, Pakistan [grant number 20-15988/NRPU/R&D/HEC/2021].

## Declaration of competing interest

The authors declare that they have no known competing financial interests or personal relationships that could have appeared to influence the work reported in this paper.
