# Supplementary material for: Short path molecular distillation of the essential oil from Pinus roxburghii oleoresin affords volatile fractions with powerful antioxidant and antimicrobial activities comparable with common synthetic agents and antimicrobials
Source: Heliyon. 2025 Jan 25;11(3):e42282. doi: 10.1016/j.heliyon.2025.e42282 (PMC11847250; doi:10.1016/j.heliyon.2025.e42282)
Supplement: Multimedia component 1 [file mmc1.docx]

**Supplementary Information**

**Short Path Molecular Distillation of the Essential Oil from *Pinus roxburghii* Oleoresin Affords Volatile Fractions with Powerful Antioxidant and Antimicrobial Activities Comparable with Common Synthetic Agents**

Muhammad Adnan Ayub^1^*, Hawraz Ibrahim M. Amin^2,3^*, Rameen Waseem^1^, Kamaran Younis M. Amin^4^, Muhammad Asif Hanif^5^, Amjad Hussain^6^, Kovan Dilawar Issa^7^, Jorge Ramírez^8^, Chabaco Armijos^8^, Muhammad Zubair^9^, Giovanni Vidari^7*^

^1^Department of Chemistry, University of Sahiwal, 57000 Sahiwal, Pakistan; [adnanayub@uosahiwal.edu.pk](mailto:adnanayub@uosahiwal.edu.pk) and [rameenwaseem422@gmail.com](mailto:rameenwaseem422@gmail.com)

^2^ Department of Chemistry, College of Science, Salahaddin University-Erbil, Erbil 44001, Iraq

^3^ Department of Medical Biochemical Analysis, Cihan University-Erbil, Erbil 44001, Iraq; Hawraz.mohammedamin@su.edu.krd

^4^ Department of Chemistry, College of Education, Salahaddin University-Erbil, Erbil 44001, Iraq; kamaran.younis@su.edu.krd

^5^ Department of Chemistry, University of Agriculture Faisalabad, Faisalabad 38000, Pakistan

^6^ Institute of Chemistry, University of Okara, Okara-56300, Punjab; Pakistan; amjadhussain@uo.edu.pk

^7^ Department of Medical Analysis, Faculty of Applied Science, Tishk International University, Erbil 44001, Iraq; [Kovan.dilawer@tiu.edu.iq](mailto:Kovan.dilawer@tiu.edu.iq) (KDI); [vidari@unipv.it](mailto:vidari@unipv.it) (GV)

^8^ Departamento de Química, Universidad Técnica Particular de Loja, Loja 110107, Ecuador; [jyramirez@utpl.edu.ec](mailto:jyramirez@utpl.edu.ec) (JR); [cparmijos@utpl.edu.ec](mailto:cparmijos@utpl.edu.ec) (CA)

^9^ Department of Chemistry, Faculty of Science, University of Gujrat, Gujrat 50700, Pakistan

* Correspondence: [adnanayub@uosahiwal.edu.pk](mailto:adnanayub@uosahiwal.edu.pk) (MAA); Hawraz.mohammedamin@su.edu.krd (HIMA); [vidari@unipv.it](mailto:vidari@unipv.it) (GV).

S1. Table 3. Refence table of standard *n*-alkanes from C_7_ to C_24_. Page 2

S2. Table 4. Retention time (tR) and calculated Linear Retention Index (LRI_calcd_)

of the components of the oil EO_4_. Page 3

S1. Table 3. Refence table of standard *n*-alkanes from C_7_ to C_24_.

|  |  | |  |  |
| --- | --- | --- | --- | --- |
| Retention time (tR) | Next tR | Denominator | Carbon Number | C N Diff |
| 0.00 | 1.30 | 1.3000 | 7 | 1 |
| 1.30 | 4.45 | 3.1500 | 8 | 1 |
| 4.45 | 7.10 | 2.6500 | 9 | 1 |
| 7.10 | 10.75 | 3.6500 | 10 | 1 |
| 10.75 | 15.00 | 4.2500 | 11 | 1 |
| 15.00 | 19.46 | 4.4600 | 12 | 1 |
| 19.46 | 23.88 | 4.4200 | 13 | 1 |
| 23.88 | 28.14 | 4.2620 | 14 | 1 |
| 28.14 | 32.22 | 4.0780 | 15 | 1 |
| 32.22 | 36.10 | 3.8800 | 16 | 1 |
| 36.10 | 39.80 | 3.7000 | 17 | 1 |
| 39.80 | 43.33 | 3.5300 | 18 | 1 |
| 43.33 | 46.69 | 3.3600 | 19 | 1 |
| 46.69 | 49.92 | 3.2275 | 20 | 1 |
| 49.92 | 53.00 | 3.0825 | 21 | 1 |
| 53.00 | 56.05 | 3.0500 | 22 | 1 |
| 56.05 | 58.99 | 2.9420 | 23 | 1 |
| 58.99 | 60.50 | 1.5080 | 24 | 1 |

S2. Table 4. Retention time (tR) and calculated Linear Retention Index (LRI_calcd_) of the components of the oil EO_4_.

| tR of EO_4_ components | LRI_calcd_ of EO_4_ components |
| --- | --- |
|  |  |
| 5.085 | 924 |
| 5.325 | 933 |
| 5.754 | 949 |
| 6.344 | 971 |
| 6.797 | 989 |
| 7.218 | 1003 |
| 7.447 | 1010 |
| 7.792 | 1019 |
| 7.967 | 1024 |
| 8.123 | 1028 |
| 8.183 | 1030 |
| 10.187 | 1085 |
| 10.727 | 1099 |
| 10.842 | 1102 |
| 12.355 | 1138 |
| 12.402 | 1139 |
| 12.533 | 1142 |
| 13.334 | 1161 |
| 14.718 | 1193 |
| 15.058 | 1201 |
| 15.162 | 1204 |
| 15.861 | 1219 |
| 18.755 | 1284 |
| 21.672 | 1350 |
| 22.368 | 1366 |
| 22.645 | 1372 |
| 23.508 | 1392 |
| 24.159 | 1407 |
| 24.551 | 1416 |
| 31.393 | 1580 |
